# An Opto-Electronic Sensor-Ring to Detect Arthropods of Significantly Different Body Sizes

**DOI:** 10.3390/s20040982

**Published:** 2020-02-12

**Authors:** Esztella Balla, Norbert Flórián, Veronika Gergócs, Laura Gránicz, Franciska Tóth, Tímea Németh, Miklós Dombos

**Affiliations:** 1 Department of Fluid Mechanics, Faculty of Mechanical Engineering, Budapest University of Technology and Economics, Bertalan Lajos utca 4-6, H-1111 Budapest, Hungary; balla@ara.bme.hu; 2 Centre for Agricultural Research, Institute for Soil Sciences and Agricultural Chemistry, Herman Ottó út 15, H-1022 Budapest, Hungary; florian.norbert@agrar.mta.hu (N.F.); gergocs.veronika@agrar.mta.hu (V.G.); granicz.laura@agrar.mta.hu (L.G.); toth.franciska@agrar.mta.hu (F.T.); nemeth.timea@agrar.mta.hu (T.N.)

**Keywords:** insect detection, infrared sensor, environmental monitoring, integrated pest management, microarthropods, Collembola, Oribatida, Acari

## Abstract

Arthropods, including pollinators and pests, have high positive and negative impacts on human well-being and the economy, and there is an increasing need to monitor their activity and population growth. The monitoring of arthropod species is a time-consuming and financially demanding process. Automatic detection can be a solution to this problem. Here, we describe the setup and operation mechanism of an infrared opto-electronic sensor-ring, which can be used for both small and large arthropods. The sensor-ring consists of 16 infrared (IR) photodiodes along a semicircle in front of an infrared LED. Using 3D printing, we constructed two types of sensor-ring: one with a wider sensing field for detection of large arthropods (flying, crawling, surface-living) in the size range of 2–35 mm; and another one with a narrower sensing field for soil microarthropods in the size range of 0.1–2 mm. We examined the detection accuracy and reliability of the two types of sensor-ring in the laboratory by using particles, and dead and living arthropods at two different sensitivity levels. For the wider sensor-ring, the 95% detectability level was reached with grain particles of 0.9 mm size. This result allowed us to detect all of the macroarthropods that were applied in the tests and that might be encountered in pest management. In the case of living microarthropods with different colors and shapes, when we used the narrower sensor-ring, we achieved the 95% detectability level at 1.1 mm, 0.9 mm, and 0.5 mm in the cases of F. candida, H. nitidus, and H. aculeifer, respectively. The unique potential of arthropod-detecting sensors lies in their real-time measurement system; the data are automatically forwarded to the server, and the end-user receives pest abundance data daily or even immediately. This technological innovation will allow us to make pest management more effective.

## 1. Introduction

Arthropods have great importance and effects on human well-being and the economy. Several species have harmful effects (e.g., pests or vectors of diseases), and others are responsible for several agricultural and natural ecosystem processes (e.g., pollinators [1] or decomposers [2]). There is an increasing need to monitor arthropods in food plants and storage [3]. One of the most critical applications of insect monitoring is in the field of integrated pest management (IPM), which aims to optimize plant protection actions on arable fields and orchards [4,5]. It is essential in plant protection to ascertain the population size and activity time of different arthropod species. However, the estimation of the population size of arthropods is currently a time-consuming and financially demanding process. In traditional arthropod monitoring, experts are needed to check the traps at least once a week in the field. If the checking frequency were lower, the accuracy of the monitoring would rapidly decrease, and in the case of pest management, the timing of spraying would not be adequate. Automatic detection and counting of arthropods could be a solution because it would significantly decrease labor inputs by reducing on-site manual checks and increasing precision due to continuous measurement [6].

For that reason, in the last decade, rapid development has been experienced in the field of arthropod detection. For detection, studies use different technical solutions, which have been reviewed in earlier papers [7,8]. Focusing on solutions that were developed to detect specimens of an arthropod species in the field using traps equipped with one kind of sensor, we can find, among others, acoustic or pseudo-acoustic sensors [9,10,11,12,13,14], opto-electronic sensors with lasers [15,16] or infrared beams [17,18,19,20,21,22], and camera devices [23,24,25,26,27].

Opto-electronic sensors, as the more traditional ones, have a long history. Several studies examine the signal and the attributes of arthropods’ wingbeats [21,28,29], and the polarization of the surface of insects [29]. Nevertheless, there are other simpler sensors which detect animals falling or flying through a sensor field [8] (Table 1). 

Arthropod species are very diverse; they can differ in body size by two orders of magnitude and have body plans adapted to many different lifestyles, for example, flying, crawling, ground-living, and soil-living. The body shape and life form of the species strongly influence the appropriate method of detection. Here, we demonstrate an infrared opto-electronic sensor-ring (IRSR) by which arthropods of significantly different sizes and shapes can be detected. We aimed to build a real-time monitoring instrument for pest management and environmental protection that could fulfill both the requirements of farming needs and larger-scale environmental policies. Our previous prototype, called EDAPHOLOG, is a monitoring device that operates with opto-electronic sensors and can detect the activity of animals living in the soil [35]. We have previously developed another type of infrared sensor for soil microarthropods that was also used in EDAPHOLOG probes [36]. Based on this prototype and other prototypes developed in the frame of the INSECTLIFE project (zoolog.hu/insectlife), we present a universal sensor-ring that fits into several types of traps.

**Figure 1:**
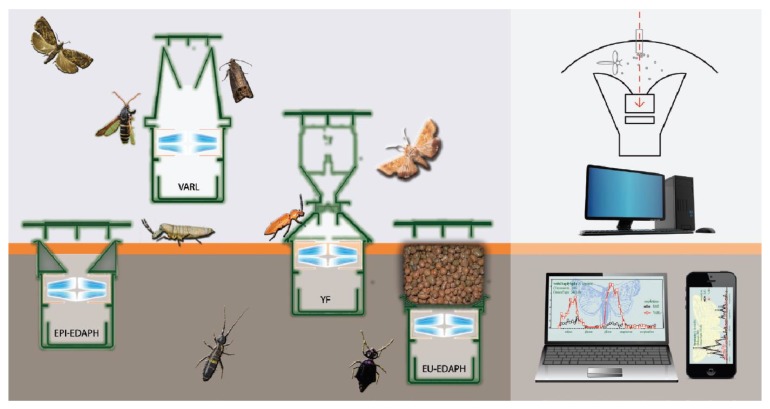
ZooLog Sensor System. Probes (sensors installed in traps) are used for flying (VARL), surface-living (YF, EPI-EDAPH), and soil-living arthropods (EU-EDAPH). CSALOMON pheromone traps and a new EDAPHOLOG trap were equipped with infrared sensor-rings. The online monitoring system consists of probes, loggers, and a central database retrievable via an internet browser. For further information about the acronymic-named trap types, see Supplement 1.

In this paper, we describe the mechanical construction, hardware setup, and precision tests of a unified opto-electronic sensor-ring, which is able to detect both flying, crawling, and ground-living arthropods of different sizes. We built the IRSR into traps already used in the CSALOMON pheromone trap family (www.csalomoncsapdak.hu) (Figure 1). The so-called VARL-type CSALOMON trap is used for flying insects (2–30 mm), KLP traps are used for crawling insects such as corn rootworms (25 mm), and YF traps are used for click beetles, which sometimes fly and sometimes move on the ground (7–13 mm). For a detailed description of the acronymic-named traps, see Supplement 1. The ZooLog Sensor System prototype consists of probes (sensors within traps), loggers, and a central database. Data are transferred via the internet, and activity patterns of arthropod populations over time are visualized and are available on any internet browser.

## 2. Materials and Methods

In Section 2.1, we present the mechanical construction and the operation of the opto-electronic infrared sensor-ring (IRSR); Section 2.2 shows the electronic construction of the IRSR; in Section 2.3, the fall detection algorithm is described, while in Section 2.4 the adjustable settings of the sensor are discussed. In Section 2.5, we present the methods of different laboratory tests on the accuracy and efficiency of the IRSR with various small objects and animals. The features of the statistical analysis applied to the laboratory tests are presented in Section 2.6.

### 2.1. Mechanical Construction and Operation of the Opto-Electronic IR Sensor-Ring

**Figure 2:**
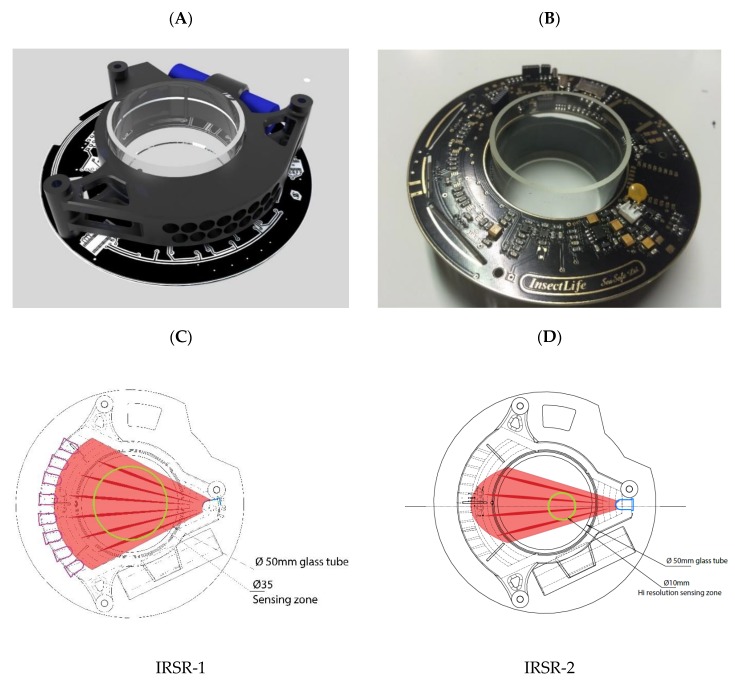
The mechanical construction of the infrared opto-electronic sensor-ring (IRSR). (**A**): whole sensor unit. (**B**): printed circuit board with a glass tube. (**C**): the draft of IRSR-1 for larger arthropods. (**D**): the draft of IRSR-2 for microarthropods.

The whole sensor unit and the printed circuit board with the glass tube are shown in Figure 2A and 2B respectively. The basis of detection is a light gate, which operates within the infrared spectrum to lower the disturbing environmental light effects. Therefore, we used infrared LEDs as a light source and photodiodes as detectors. Objects crossing the detection field result in lower light intensity detection on the sensors. The amount of shading depends on the size, shape, transparency, position, and orientation of the object.

Considering the shape and functioning of the traps, we constructed a detection field on the cross-section of a tube. Any arthropod entering the trap has to fly in or fall into this glass tube. The diameter of the catching part of the trap tube is 35 mm in the VARL, KLP, and YF traps and 10 mm in EDAPHOLOG (see the yellow circle in Figure 2C). The infrared detection field was constructed by using 16 infrared (IR) photodiodes along a semicircle (Figure 2C), in front of the infrared LED. Both the photodiodes and the LEDs were placed in two rows, offset from each other, to achieve a denser IR detection field. High sensitivity was reached using two infrared LED light sources, which were positioned vertically, and their emission direction was shifted by a half-beam angle compared to each other. With this method, the angle in which effective sensing can be achieved was increased, and the light intensity in the measurement section became more homogeneous compared to using only one light source. BPV10NF-type photodiodes were used with focusing optics. This arrangement made it possible to detect arthropods of different body sizes by up to two orders of magnitude. 

The first three trap types are used for bigger arthropods with a body size of 2–35 mm. For this, we designed the IRSR-1 (Figure 2C) with a larger but less sensitive detection field (yellow circle in Figure 2C). The EDAPHOLOG-like traps (EU- and EPI-EDAPH) are used for microarthropods (0.1–2 mm), and for this we constructed the IRSR-2 with a much denser and therefore more sensitive detection field (Figure 2D). Higher sensitivity could be achieved by placing the active detection field closer to the light source and photodiodes closer to each other. The height (longitudinal section of the tube) of the IRSR was set to 15 mm. Photodiodes and IR LEDs were installed in a black, 3D-printed plastic frame. For decreasing environmental light noise, a banded surface was 3D-printed on the inside of the IRSR, which absorbs light coming through the glass tube. Arthropods have to pass through a glass tube with a diameter of 50 mm and a length of 38 mm to get into the IRSR. The blueprint of the 3D-printed frame is available in Supplement 1.

### 2.2. Sensor Electronics

Sensor electronics can be divided into four main parts: power supply; analog amplifier; CPU unit; and communication system. Supplement 1 presents the detailed schematics of the sensor electronics, including the central microcontroller unit, the photodiode receivers, and the communication unit.

The power supply is based on 3.6 V Li-ion accumulators. The average current consumption of the whole system is 5.5 mA, and it is 6 mA at maximum. The current consumption for GSM communication can reach 1 A for 0.5 ms periods; however, this only amounts to a few percent of the operating time. The system can operate for between two weeks and one month with a single charge. If there is a demand for a longer operational time, solar cells can be used for power supply.

The MCP6141-type operational amplifier was chosen to be included in the circuit, as its current consumption is only 600 nA and it operates stably in at least tenfold amplification connection. Its gain-bandwidth product is 100 kHz. The first level of the photodiode amplifier is set to 15x amplification with the MCP6141, so an upper-frequency limit of approximately 6 kHz is appropriate, and there is no need for a separate low-pass filter. The second stage is connected through a high-pass filter of around 5 Hz corner frequency, with approximately 30x amplification. Here, a higher bandwidth operational amplifier must be chosen because of higher amplification and stronger output, since the A/D converter’s multiplexer operates well only at low impedance. The applied operational amplifier is an MCP607 with 600 kHz bandwidth, which can be used within the full supply voltage range; it has a low offset voltage, and its current consumption is 60 µA. The main characteristics of the amplifiers and the power supply are summarized in Table 2.

The operational amplifiers of the photodiodes work on a low signal level; thus, they are particularly sensitive to the power supply voltage noise. Because of this, these amplifiers receive a separate power supply from an independent power circuit.

### 2.3. Detection Algorithm

The amplified voltage signal of the sensor contains high time-constant noise. This was filtered out with the use of the moving average technique. The sampling frequency of the A/D is 3.2 kHz per channel. Thirty-two samples are included in the moving average, making the system more sensitive to changes with time constants of less than 10 ms. According to the test results, the time constant of arthropod falls is around 5 ms. The way of calculating the consequent averages is to replace the oldest sample with the newest one. With this method, the latest samples become the parts of the average calculation with the highest weights, while previous samples have lower weights. The mean determines the baseline to which differences are compared. 

The sensing program continuously monitors the channels and compares the measured signal to the computed baseline level. If the difference between the signal and the baseline value exceeds the set detection threshold level, then the detection begins. During the detection period, the detection amplitude is calculated by summing the signal values above the threshold level. The detection ends when the measured signal remains below the detection threshold level for a specific length of time. After the detection is closed, the summarized data are normalized and stored by the program along with the sensing data.

Normalization is performed by calculating the average of the summarized data; the sum is divided by the number of included samples. The value of the divisor is rounded to the nearest round binary number to increase calculation speed. With this optimization, the program can carry out the task by a shift operation in one clock signal, instead of the time-consuming division operation. The maximum of the error introduced by this method is 50%, which is acceptable, as the measurements are already contaminated with noise in the same order of magnitude due to other sources of noise. The flow charts presenting the baseline calculation and the detection algorithm are included in Supplement 1.

Data are stored on an EEProm with a capacity of 8MB, which is connected to the CPU through an SPI bus. With this capacity, more than 500 detections per day can be stored for more than half a year. The microcontroller can send the data to the central server through GPRS communication. Previously used EEProm spaces will only be reused when storage already went through all address spaces. With this method, ’flash’ memory wear remains equally distributed, which is necessary as such memory types can only be rewritten a limited number of times.

### 2.4. Settings of the Detection Procedure

The parameters in the detection algorithm can be set according to the target species having a given body size, making the sensor suitable for more trapping devices. From the testing point of view, the detection threshold (sensitivity threshold; ST) is the most crucial parameter. Based on preliminary sensitivity tests of the circuit board for arthropods bigger than 1 mm, this parameter (ST) was set to 100. For tiny arthropods, the detection threshold was set to ST = 40, at which, however, environmental noise might increase. There were many other parameters, for example, parameters related to the data communication and the control of other parts of the probe, but these parameters are not detailed here.

### 2.5. Laboratory Testing of Detection Efficiency

We measured the detection efficiency of IRSR-1 and IRSR-2 in three laboratory tests by using (1) grains with known sizes, (2) dead arthropods larger than 1 mm (flying and crawling), and (3) living microarthropods. In these experiments, we evaluated the accuracy and precision of the sensors at detection thresholds set to ST = 40 and ST = 100.

#### 2.5.1. Detectability Investigations with Particles

In the first test, we dropped stone grains into both IRSR-1 and IRSR-2 at the detection threshold of 100. We measured the size of the grains in digital images taken of the particles in a stereomicroscope. Then, we categorized the grains into six size categories between 0.2 and 1.8 mm (ranges: 0.2 mm). Following size measurements, we dropped approximately 20 particles of each group into the sensor-ring and recorded whether they were detected or not.

#### 2.5.2. Detectability Investigations with Arthropods

##### Detection Accuracy of IRSR-1 for Large Arthropods

We used large dead adult specimens of nine arthropod species for the evaluation of the detection accuracy of IRSR-1 (for the species list, see Table 2). These arthropods were previously captured with CSALOMON pheromone traps in an orchard at our test area at Julianna major (Budapest, Hungary). We had to use dead animals because dropping specimens of flying species was not feasible and we could not keep these animals alive in the laboratory. For the determination of the lower sensitivity range of IRSR-1, we applied living specimens of two further microarthropod species: *Heteromurus nitidus* Templeton (Collembola: Entomobryidae) and *Folsomia candida* Willem (Collembola: Isotomidae) from laboratory culture. A minimum of 20 individuals of each species were dropped into the sensor-ring separately, in the same way as in the experiment with particles. Due to their different sizes, these microarthropods were of various ages. 

##### Detection Accuracy of IRSR-2 for Small Arthropods

The detectability of soil-dwelling and surface-living invertebrates varies with different body shapes, sizes, and appendages (e.g., hairs, scales), which was already observed in our previous study [35]. For determining the detection accuracy of the new sensor-ring, we dropped living microarthropod individuals into IRSR-2 at a detection threshold of ST = 40. Three species kept in the laboratory were used: two collembolans (*H. nitidus*, *F. candida*) and a mite species (*Hypoaspis aculeifer* Canestrini (Mesostigmata: Laelapidae)). Each specimen was used only once (430 specimens altogether), and we recorded their detectability (yes or no). We determined the body sizes of the specimens by using a stereomicroscope before the measurement, and their sizes ranged from 0.28 to 2.84 mm.

##### Sensitivity Test of IRSR-2

To measure the differences in the accuracy at detection thresholds of ST = 40 and ST = 100, we investigated living microarthropods (*H. nitidus*). A total of 299 specimens were dropped into IRSR-2, and detection was then recorded and detectability was calculated.

### 2.6. Statistics

We analyzed the detectability of particles and the different arthropod groups by using binomial generalized linear models (GLMs). In the models, the recorded detection or the lack of it was used as the binomial response variable; species and body size (measured manually under a stereomicroscope) were used as explanatory variables. A logistic distribution function was fitted between the measured size ranges and the predicted values from the results of the GLMs. The models were calculated with R program version 3.6.1 [39].

## 3. Results

### 3.1. Detectability of Particles Using IRSR-1 and IRSR-2

**Figure 3:**
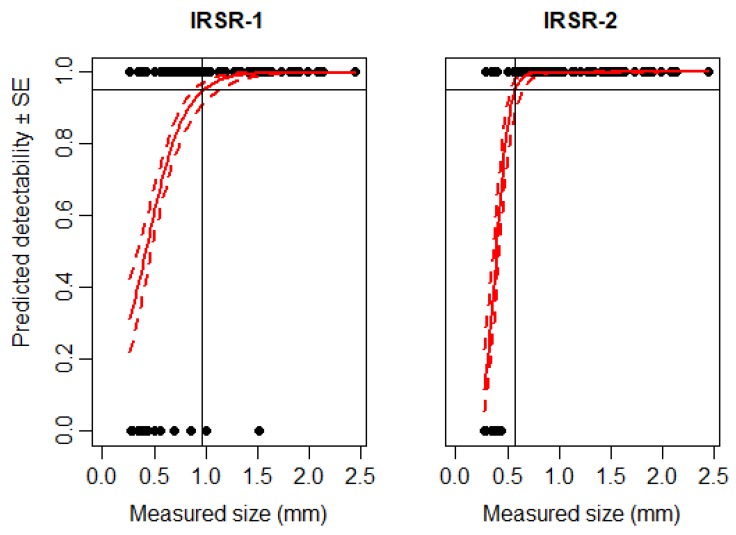
Expected detectability (continuous red line) ± SE (dashed red line) of grain particles according to particle size using IRSR-1 and IRSR-2. A binomial generalized linear model (GLM) was used to estimate detectability. The 95% detection level is shown by the continuous black line. The detection threshold of the sensors was set to sensitivity threshold (ST) = 100.

The 95% detectability level of IRSR-1 was reached with grain particles of 0.9 mm size, while this parameter was lower (0.6 mm) in IRSR-2 (Figure 3). By using bigger particles, sensor-rings performed with 100% accuracy, and only one piece of grain particle (size: 1.5 mm) was not detected in IRSR-1.

### 3.2. Detectability of Small Microarthropods Using IRSR-2

**Figure 4:**
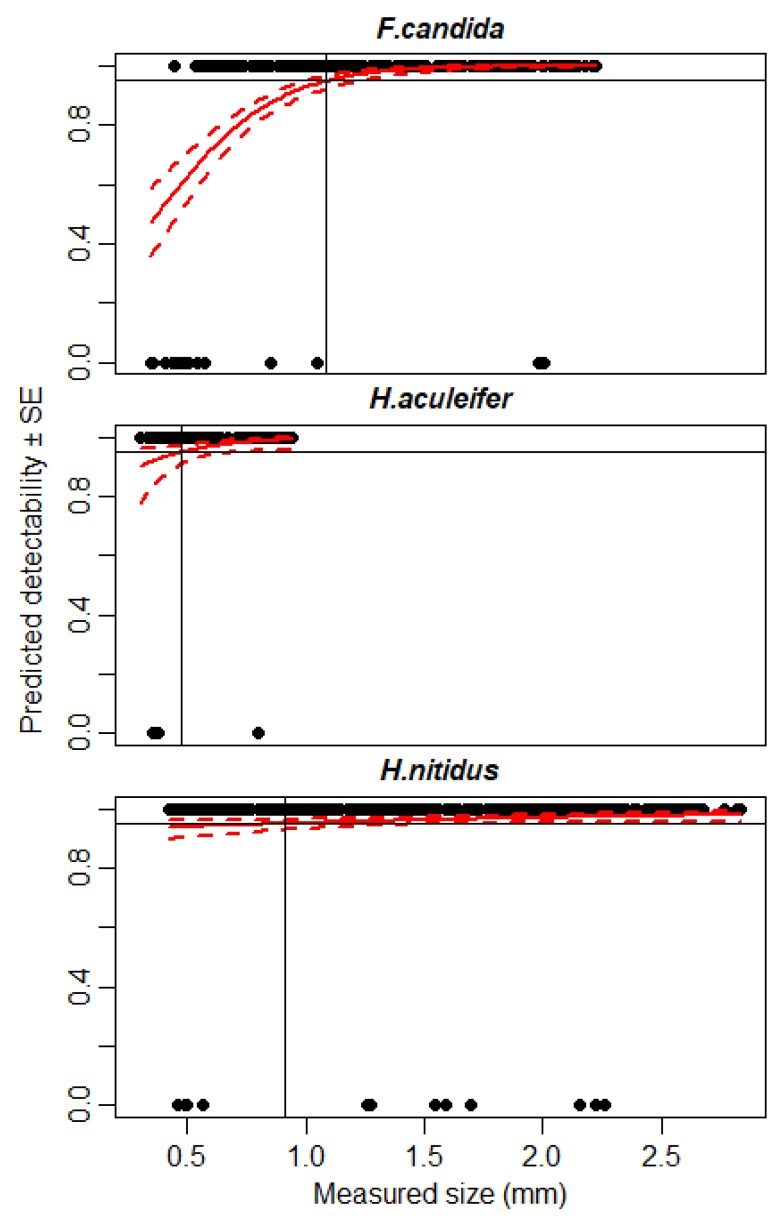
Expected detectability (continuous red line) ± SE (dashed red line) of three microarthropod species: *F. candida*, *H. nitidus*, and *H. aculeifer*. A binomial GLM was used to estimate detectability. The 95% detection level is shown by the continuous black line. The detection threshold of the sensors was set to ST = 40.

Microarthropod specimens of 0.28–2.84 mm were used in this test. By using the IRSR-2 sensor-ring at a detection threshold of ST = 40, we reached 95% detectability at 1.1 mm in the case of *F. candida*, 0.9 mm for *H. nitidus*, and 0.5 mm for *H. aculeifer* (Figure 4). Detection failures related to bigger specimens occurred mostly for the two Collembola species (*F. candida*, *H. nitidus*), which could be due to their opaque or shimmering body surface.

### 3.3. Sensitivity Analysis of IRSR-2

**Figure 5:**
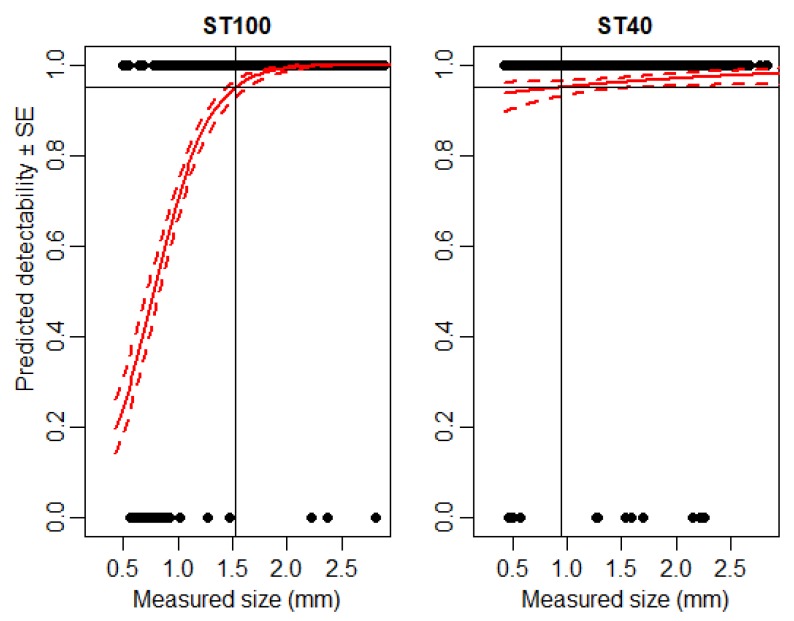
Expected detectability (continuous red line) ± SE (dashed red line) of the Collembola *H. nitidus* using IRSR-2 at two different detection thresholds (a higher sensitivity, ST = 40, was set compared to ST = 100). A binomial GLM was used to estimate detectability. The 95% detection level is shown by the continuous black line.

The sensitivity of IRSR-2 was investigated at two different detection thresholds (ST = 40 and 100). Here, we dropped living springtails (*H. nitidus*) into the sensor-ring. At ST = 40, IRSR-2 detected the smaller animals (1–1.5 mm) more effectively than at ST = 100 (Figure 5).

### 3.4. Detectability of Large Arthropods Using IRSR-1

IRSR-1 detected all of the large arthropod individuals ranging from 1.40 to 23.96 mm in body size (Table 3). Smaller microarthropods of sizes between 0.26 and 2.38 mm were detected at a considerably lower percentage. These two microarthropod species were only used to indicate the limits of the detection accuracy of IRSR-1, which was designed to detect arthropods larger than 2 mm.

## 4. Discussion

In this paper, we showed the performance of a new sensor-ring applying infrared photoelectric sensor technology to detect arthropods of significantly different body sizes. Our sensor-rings detect arthropods flying or falling through the sensor field. We developed the IRSR-1 sensor for detecting macroinvertebrates, while IRSR-2 with a narrower sensor field can detect arthropods of small size. These sensors are uniform in shape and can be built into several types. We aimed to develop a uniform infrared sensor-ring that can be used for several arthropod species. Most of the previously developed sensors were designed and tested for one species or a specific arthropod group. However, in practical pest control and IPM, several pests appear simultaneously and they should be monitored in parallel. Some of the sensors were placed in a regular insect trap (see the second column in Table 1) that fulfills this practical requirement. The current proposed sensor-ring can installed into the CSALOMON trap family, into VARL-type traps for flying insects (used for more than one hundred pest species in orchards and vineyards), into KLP-type traps for crawling insects (used for imagos of corn rootworm species in cornfields), and into YF traps for (partly) surface-living insects (used for click beetles in wheatfields). Therefore, real-time information can be obtained about the activity-density of a wide range of arthropods, including flying, crawling, surface-living, or ground-dwelling arthropods under field conditions. Due to their low energy consumption, these sensors can be used in remote, less accessible places for long periods of time.

We developed the IRSR-1 sensor-ring for larger (>2 mm) macroarthropods such as moths (e.g., pests in orchards) or beetles (e.g., click beetles/wireworms in cereal fields, corn rootworms in cornfields). With its larger sensor field (diameter: 35 mm), IRSR-1 can detect all of the macroarthropod pests caught by a traditional funnel or pitfall trap used in pest monitoring. We found that the detectability reached 100% at the body size range of the target species; therefore, IRSR-1 is accurate for macroarthropod monitoring.

Regarding smaller objects, in the first experiment, the IRSR-1 sensor reached 95% detectability at about 1 mm, and the IRSR-2 at 0.6 mm, respectively. These particles were similar in shape and color. However, the accuracy of detection depends on the size, shape, and even on the movement pattern of the given specimen [11]. In our previous work [35,36], we found that the morphology of arthropods (pigmentation, color, hairs, and appendages) influenced the detectability. These differences in detectability could be seen in the current experiments as well.

The earlier version of our infrared sensor [35,36] was built for soil microarthropods with small body sizes and reached a 90%–95% detection rate only at 1 mm body size. In contrast, the new IRSR-2 sensor-ring detected smaller (>0.6 mm) individuals with high accuracy. In the earlier infrared sensor, plano-convex lenses were used to increase sensitivity; however, in the current sensor-ring, we could achieve higher sensitivity without lenses. This could be achieved by using 16 IR photodiodes along a semicircle, in front of the infrared LED. Both the photodiodes and the LEDs were placed in two rows, offset from each other, to achieve a denser IR detection field. This result highlights that the geometric design of the sensor array is crucial for the sensitivity of the probe. Detection accuracy did not reach 100%, even with animals larger than 1.2 mm, due to some undetected individuals. Detecting living springtails is complicated because they often bend their appendages while they are falling, and these changes in body shape influence the probability of positive detection. This is probably why the detection accuracy is lower for living microarthropods than for stone grains of the same size.

We found differences in detectability at different sensitivity threshold levels (ST = 40 and ST = 100). Setting the threshold level to ST = 40 resulted in higher detectability at lower body sizes; however, it produced more false detections, lowering the measurement precision and accuracy. White noise might come from environmental lights. Therefore, the more sensitive ST = 40 setting might be more appropriate for a controlled environment such as laboratory experiments, whereas the ST = 100 setting is less sensitive, and therefore, more adequate for outdoor studies.

As Potamitis, Rigakis and Fysarakis [19] summarized, an opto-electronic sensor only records events in which the path between the receiver and the emitter is interrupted; the sensor is vulnerable in the field and has a high signal-to-noise ratio. Regarding the design of opto-electronic sensors and trapping probes in Table 1, the earliest probes were established to detect insect larvae in stored products. Accurate detection of these wingless insects was relatively easy, since usually there are no other arthropods entering the trap and the exemplars usually fall down into the probe tube. The idea of detection of microarthropods is similar to that of this first type of probe; however, microarthropods are very small animals, which is a challenge for IR sensoring. IR sensors can also provide a rough estimation of the body sizes of the detected specimens. Concerning flying insects, mostly fruitflies were detected by using opto-electronic sensors in McPhail traps. They had an evolution from the simpler infrared beams to the more sophisticated ones (see in Table 1: Potamitis, Rigakis, Vidakis, Petousis and Weber [20]), which was based on the attributes of arthropods’ wingbeats [21,28,29]. The species selectivity is a very big step in automatic monitoring of arthropods, since both practical IPM experts and field ecologists are interested in target pest species or the population dynamics of a particular species. Wingbeat sensoring was tested only in McPhail traps, in which small-sized fruitflies can fly through the sensor field within the probe. Opto-acoustic sensoring can be obtained in our probes too; however, it is beyond the scope of this paper. Opto-acoustic sensors might provide a great possibility to detect flying insects, but further standardization of the IR sensor design might be needed to make them more widely available on the IPM market. Cameras and photoshooting are the competing methods, and they function in two ways: (1) yellow sticky traps are installed in the field, and images are analyzed according to species; and (2) fast cameras are used in the entrances of the traps. Sticky cards are less usable in the field, and only a limited number of species can be caught properly. Fast cameras with real-time, on-board image analysis might be of interest, but they still have a high electronic power consumption hindering their usage in the field. IR sensors could also be used, in this case as a ‘watchdog’. When an arthropod falls into the trap, the IRSR detects it, then activates the camera, which takes a photo or film of the individual of the target species. Thus, current consumption could be radically lower compared to a continuous operation.

The unique potential of arthropod-detecting sensors lies in their real-time measurement system: the data are automatically forwarded to the server, and the end-user receives data daily or even immediately. This technological innovation will allow us to make pest control more effective. By applying fewer pesticides or applying them in a more effective way, a reduction of harmful environmental impacts on the agro-system can be achieved. Moreover, this tool itself can promote the use of environmentally friendly integrated pest management methodologies since the monitoring of pests becomes faster, resulting in more accurate pest control, and also becomes cheaper and more cost-effective.

## 5. Conclusions

Opto-electronic and opto-acoustic sensoring provide great possibilities to detect arthropods in the field. We designed and tested infrared sensor-rings to detect arthropods belonging to various taxonomic groups and therefore with very different body sizes. We constructed two types of sensor-ring with the same mechanical frame: one with a wider sensing field for detection of large arthropods in the size range of 2–35 mm, and another one with a denser, but narrower, sensing field for soil microarthropods in the size range of 0.1–2 mm. These sensor-rings detected the target species accurately and can be used in practical IPM and field ecology. These sensor-rings will be used and tested in the commercially available CSALOMON trap family for capturing flying, crawling and surface-living arthropods in pest control.

## Figures and Tables

**Table 1:** Opto-electronic sensors used for the detection of arthropods in the field. Here, we reviewed only sensor constructions that were installed into traps for field monitoring.

Target Species	Probe/Sensor	Body Size (mm)/ Species Selectivity	Accuracy/ Selectivity of Trapping	Publication/ Tests (F: field, L: laboratory)
**Flying insects**				
Mediterranean fruit fly [*Ceratitis capitate* Wiedemann (Diptera: Tephritidae)]	IR sensors placed in a commercial medfly trap	3.5–5	--- pheromone	[30] F
Oriental fruit fly [*Bactrocera dorsalis*, Hendel (Diptera: Tephritidae)]	Double-counting IR sensors in a trapping tube placed in a bottle	7–8	--- pheromone	[18] F
Mediterranean fruit fly [*Ceratitis capitate* Wiedemann (Diptera: Tephritidae)]	Imagos of insects are attracted and narcotized by toxins, then counted by IR sensors	3.5–5	88%–100% pheromone	[31] F, L
Olive fruit fly [*Bactrocera oleae* Rossi (Diptera: Tephritidae)]	Opto-acoustic sensor and IR sensors in a McPhail trap; the wingbeat recording is based on the interruption of the emitted light	0.7–1.2 species-specific	90%–96% detailed accuracy report pheromone	[19,32] L
Fruit flies: Olive fruit fly (*B. oleae*) Mediterranean fruit fly (*C. capitate*) Oriental fruit fly (*B. dorsalis*)	IR sensor lines in a McPhail trap, opto-acoustics, with Fast Fourier Transformation analysis	0.7–8 species-specific	90%–96% detailed accuracy report pheromone	[33] F, L
Fruit flies: Olive fruit fly (*B. oleae*) Mediterranean fruit fly (*C. capitate*) Oriental fruit fly (*B. dorsalis*)	Wingbeat sensing based on Fresnel optics, stereo wingbeat recorder, equipped in a McPhail trap	0.7–8 species-specific	98%–99% pheromone	[20] F
Fruit flies: South American fruit fly [*Anastrepha fraterculus* Wiedemann (Diptera: Tephritidae)]; West Indian fruit fly [*Anastrepha obliqua* Marquart (Diptera: Tephritidae)]; South American cucurbit fruit fly [*Anastrepha grandis* Marquart (Diptera: Tephritidae)]; *C. capitate*; Carambola fruit fly [*Bactrocera carambolae* Drew and Hancock (Diptera: Tephritidae)]	McPhail trap with an opto-acoustic sensor line;	0.7–5 species-specific	simulation only	[34] L
Mosquitos, house fly, and fruit flies: *Culex tarsalis* Coquilett (Diptera: Culicidae); *Culex quinquefasciatus* Say (Diptera: Culicidae); *Culex stigmatosoma* Dyar (Diptera: Culicidae); *Aedes aegypti* Linnaeus (Diptera: Culicidae); *Musca domestica* Linnaeus (Diptera: Muscidae); *Drosophyla simulans* Sturtevant (Diptera: Drosophilidae)	Phototransistor array with laser beams; species classification according to wingbeat diversity analyzed by Bayesian network	4–15	80%–98%	[9] L
*Drosophila suzukii* (Diptera: Drosophilidae); *Drosophila melanogaster* Meigen (Diptera: Drosophilidae); Zaprionus sp. (Diptera: Drosophilidae)	Multispectral sensor, backscattered light recorder	4–7	-- pheromone	[21] L
**Wingless arthropods**				[22]
Tobacco cutworm larvae: *Spodoptera litura* Fabricius (Lepidoptera: Noctuidae)	Insect larvae are detected when they move down/fall in the trap tube. Two IR sensors (double-counting)	2.3–32	80%–90% pheromone	L, F
Soil microarthropods: Collembola, Acari	Soil microarthropods are caught in a pitfall-like trap, they fall in a small tube and are detected cross-sectionally by one IR sensor through two plano-convex optical lenses	0.5–10 body size differentiation	80%–100% no pheromone	[35,36] L, F
Four stored-product species: Flat grain beetle [*Cryptolestes pusillus* Schönherr (Coleoptera: Laemophloeidae)]; Sawtoothed grain beetle [*Oryzaephilus surinamensis* (Linnaeus, 1758) (Coleoptera: Silvanidae)]; Red flour beetle [*Tribolium castaneum* Herbst (Insecta: Coleoptera: Tenebrionidae)]; Rice weevil [*Sitophilus oryzae* Linnaeus (Coleoptera: Curculionidae)]	Trap tubes are placed in stored products equipped with one IR phototransistor	2–3	no data no pheromone	[37] L [17] F
Termites: *Reticulitermes lucifugus* Rossi (Blattodea: Rhinotermitida)	Low-power light sensor (white insects on a black background)	3–4		[38] L

**Table 2:** The main characteristics of the amplifiers and the power supply.

Amplifier	Consumption	Gain-Bandwidth Product	Amplification	Power Supply
MCP6141	600 nA	100 kHz	15x	3.6 V Li-ion accumulator
MCP607	60 µA	600 kHz	30x	

**Table 3:** Detection accuracy of the IRSR-1 sensor using dead macroarthropods and living microarthropods.

Species	Mean Size (mm)	Min. Size (mm)	Max. Size (mm)	Detection
*Hypoaspis aculeifer* (Acari)	1.32	0.26	0.92	40%
*Heteromurus nitidus* (Collembola)	1.34	0.58	2.38	83%
*Meligethes aeneus* (Coleoptera)	2.37	1.40	2.40	95%
*Cameraria ohridella* (Lepidoptera)	2.77	2.18	3.19	100%
*Diabrotica virgifera* (Coleoptera)	5.16	4.53	6.30	100%
*Agriotes ustulatus* (Coleoptera)	8.06	7.29	10.21	100%
*Ephestia kuehniella* (Lepidoptera)	8.75	6.35	10.60	100%
*Epicometis hirta* (Coleoptera)	10.53	9.13	11.70	100%
*Operophtera brumata* (Lepidoptera)	15.97	14.14	19.28	100%
*Cetonia aurata* (Coleoptera)	16.77	14.43	20.09	100%
*Autographa gamma* (Lepidoptera)	21.67	15.77	23.94	100%

## Supplementary Materials

The following are available online at https://www.mdpi.com/1424-8220/20/4/982/s1.
