# Supplementary material for: An Opto-Electronic Sensor-Ring to Detect Arthropods of Significantly Different Body Sizes
Source: Sensors (Basel). 2020 Feb 12;20(4):982. doi: 10.3390/s20040982 (PMC7070424; doi:10.3390/s20040982)
Supplement: Supplementary file 1 [file sensors-20-00982-s001.pdf]

Supplement 1

A) Trap types into which the new sensors are planned to build in

Cumulatively there are five main trap types (Table 1) into which we planned to insert the IRSR-1 and IRSR-2 sensors. Two trap types were developed by our team (EPIEDAPH, EUEDAPH), while the frames of the pheromone trap constructions (VARL, KLP, Yf) were developed by the Zoology Department of the Plant Protection Institute (Centre for Agricultural Research, Hungary). These last ones are called CSALOMON® pheromone trap family and are being developed since 1993 (http://csalomontraps.com/). The pheromone traps are used to detect the emergence of pest species and we plan to install our new sensors into these traps, as well.

Table 1. Summary of the traps types

Trap type	Target arthropod groups
VARL	Flying insects, mostly used on trees
KLP	Crawling insects, especially western corn rootworm (Diabrotica virgifera)
Yf	Click beetles
EPIEDAPH	Ground living microarthropods
EUEDAPH	Soil-dwelling microarthropods

The VARL trap type (Figure 1) is appropriate for detection, quantitative monitoring and mass trapping of Lepidopteran species, such as small leafminers (*Lithocolletidae*), and for bigger sized moth species as cutworms (*Noctuidae*). Usually their size vary between 0.5-3 cm, and their wingspan is roughly similar to their body size. VARL is a plastic funnel trap, insects are attracted by species-specific pheromone sticks, and flying through a funnel into a sample container. The VARL traps also need a separation tool, which moves the animals in electric or mechanical way right after the detection, e.g., preventing the fallen insects to get back to the sensing area.

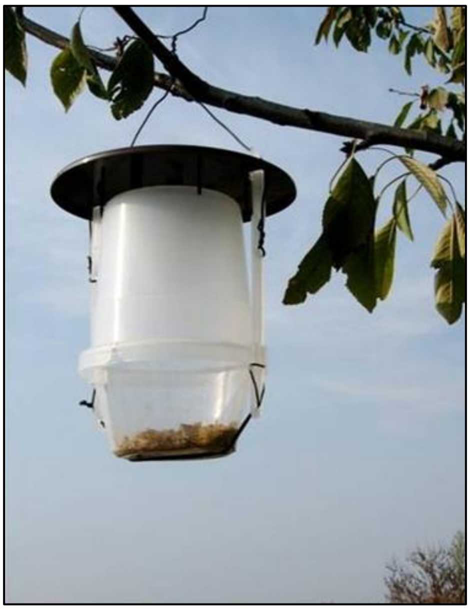

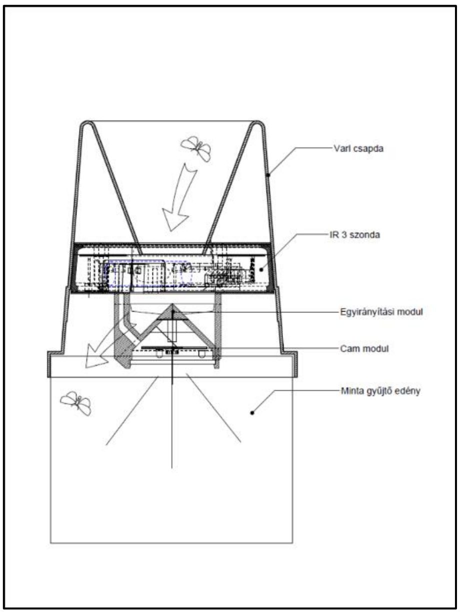

Figure 1. VARL CSALOMON trap and the draft of our planned device

The Yf (YATLORf) type trap (Figure 2) catches crawling and flying insects. It is especially efficient for catching click beetles (*Coleoptera*, *Elateridae*). The trap is effective for studying daily and seasonal activity patterns. This type of trap also uses pheromone to attract males. We plan to build our sensor-ring under the Yf trap. When the click beetles are falling into the trap, IR ring can detect it.

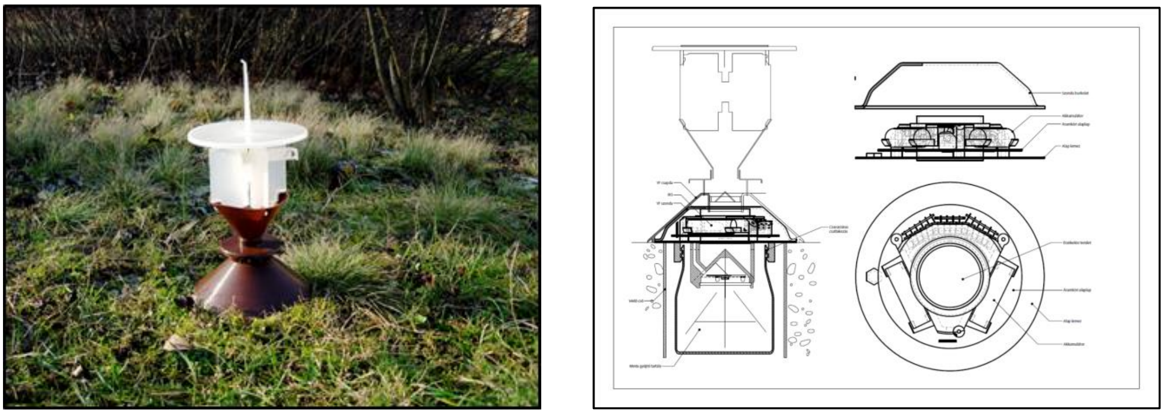

Figure 2. Yf CSALOMON trap and the draft of our planned device

There are insect groups climbing up onto the plants. This behaviour is specific for example to Western corn rootworm (*Diabrotica v. virgifera*), cabbage flea beetles (*Phyllotreta spp.)* or rape weevils (*Ceutorrhynchus assimilis* and related species). For these insects, the so called KLP traps (Figure 3) are used. In traditional CSALOMON KLP trap, the attracted insects crawl up on the vertical plate, get into the funnel trap and cannot escape. We plan to modify the probe, where the entrance of the trap is the same, but there are sensors for count the insects when they are already caught and fall down in the transparent container.

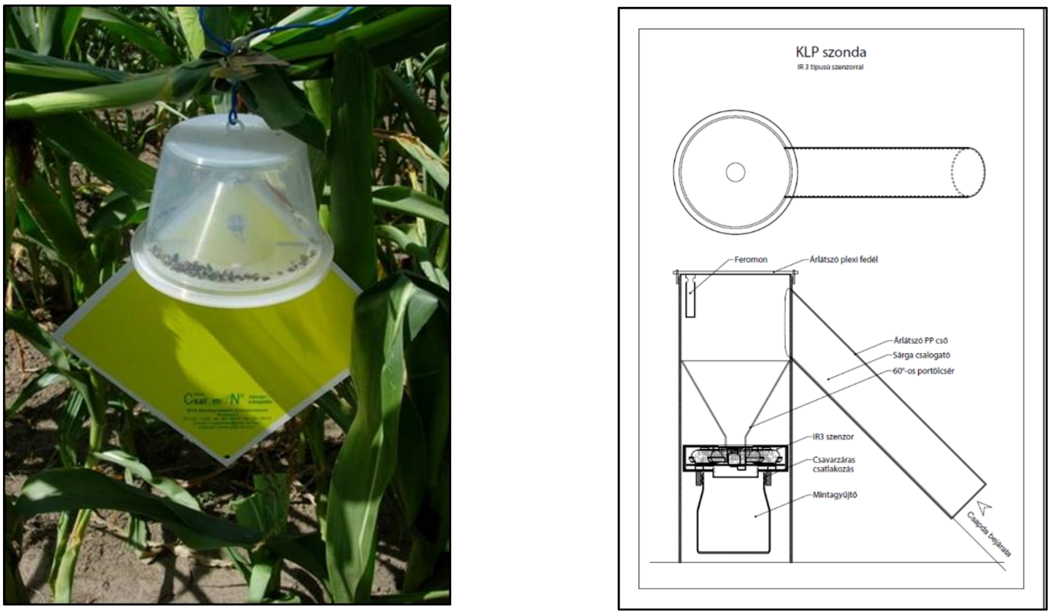

Figure 3. KLP CSALOMON trap and the draft of our planned device

Furthermore, we are developing probes for pitfall traps. It is a trapping pit for surface-living arthropods. Pitfall traps are mainly used for ecology studies and ecological pest control. We plan to detect animals when they fall into the trap.

The EPI-EDAPH trap (Fig. 4) is almost identical to the YF trap, the difference between them is the part designed for click beetles on the soil surface is missing. Instead of it, we plan to place a funnel which is sunk into the soil until the ground level.

EU-EDAPH trap (Fig. 4) is planned to design for collecting motile, ground-dwelling arthropods that are active in the upper 15 cm layer of the soil where invertebrate abundance is the highest. The sampling technique will be very similar to the methodology applied in pitfall traps. The trap will be sunk into to soil, with a 15-cm-long mesh tube, attached to the upper part of the metal tube. This is the point of entry for soil fauna into the trap. Ground-dwelling arthropods (between 0.3-10 mm) entering the trap are unable to escape, they are then captured and preserved in 70% alcohol.

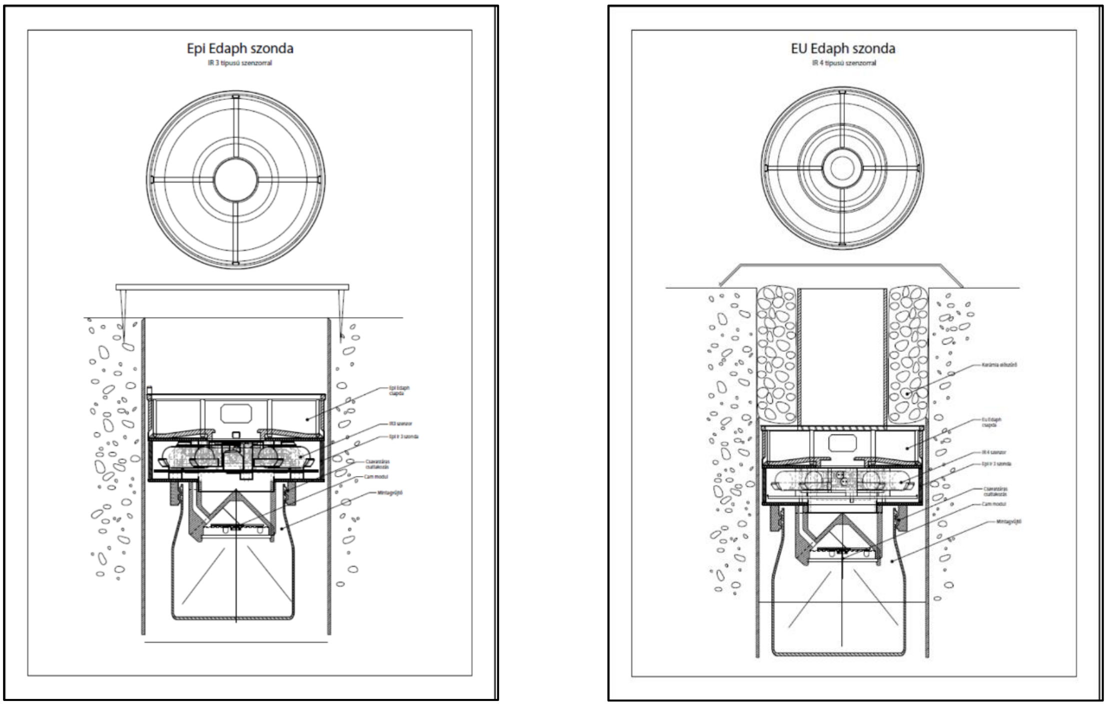

Figure 4. The drafts of the planned devices of EPI-EDAPH and EU-EDAPH traps

B) **Blueprint of the 3D printed frame of the infrared sensor-rings**

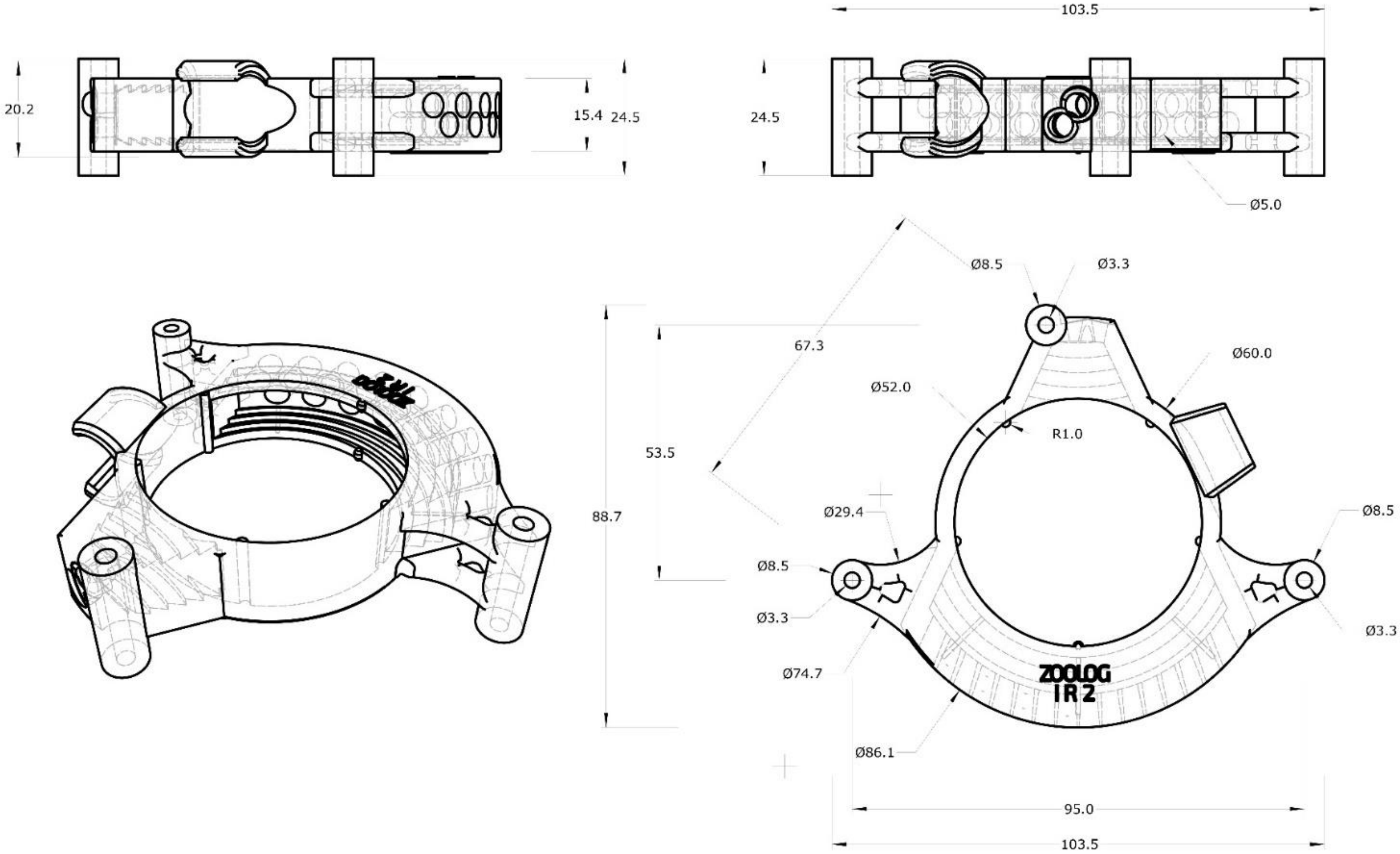

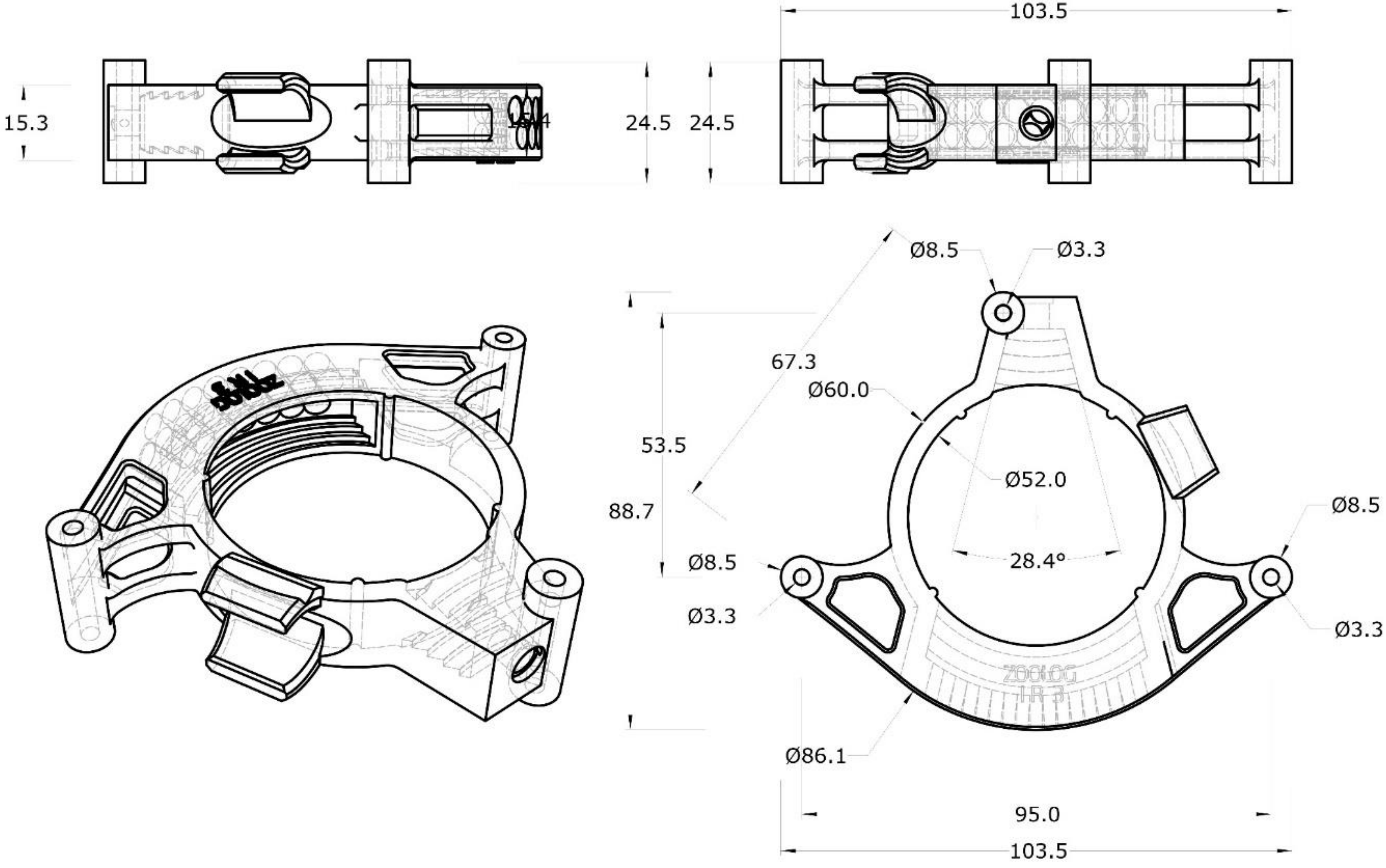

103.5
15.3
24.5
24.5
Ø8.5
Ø3.3
67.3
Ø60.0
53.5
Ø52.0
88.7
28.4°
Ø8.5
Ø8.5
Ø3.3
Ø3.3
Ø86.1
95.0
103.5

C) Schematics of the electronics and flow charts of the sensor operation

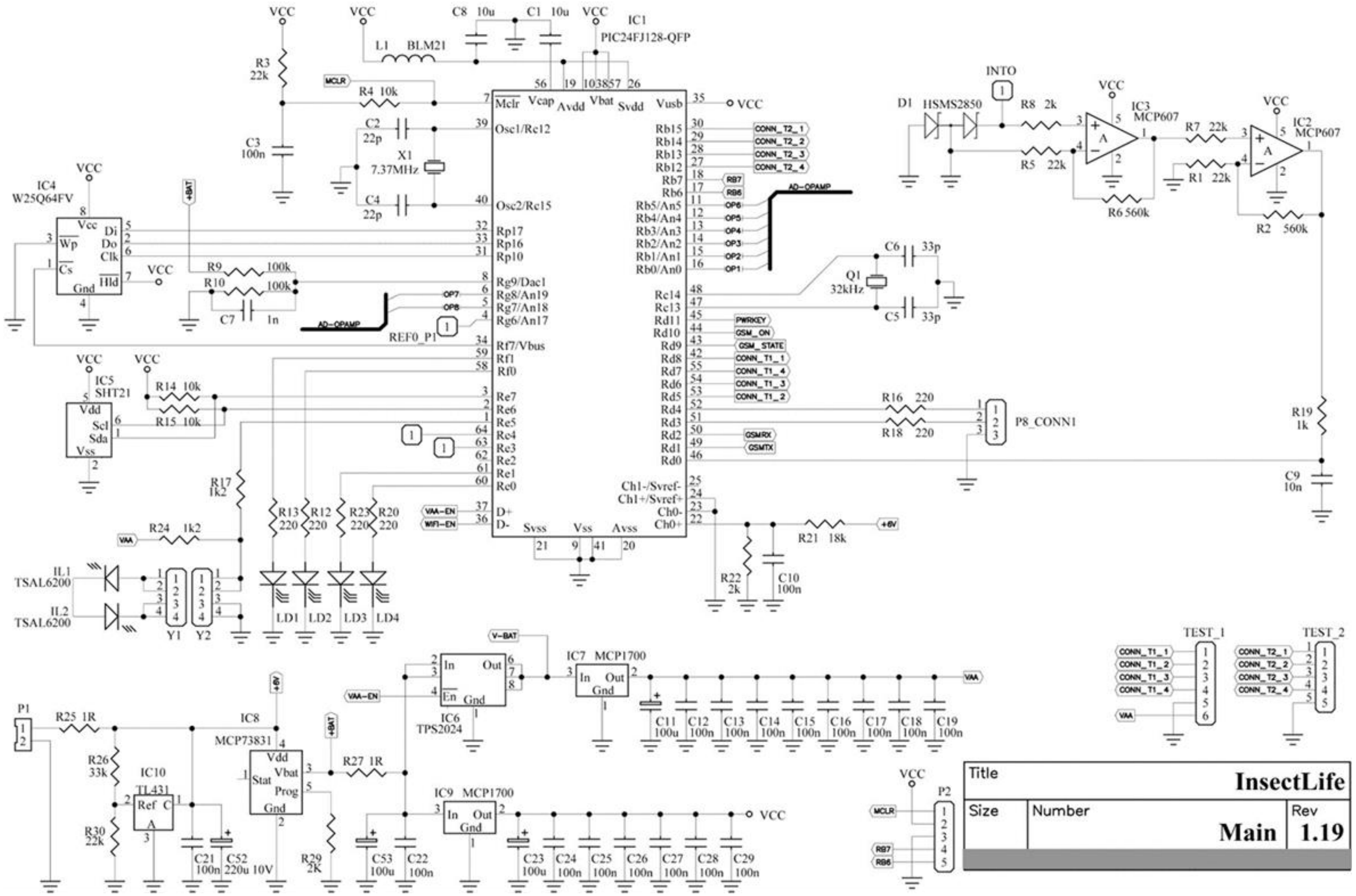

Figure 1. Microcontroller unit

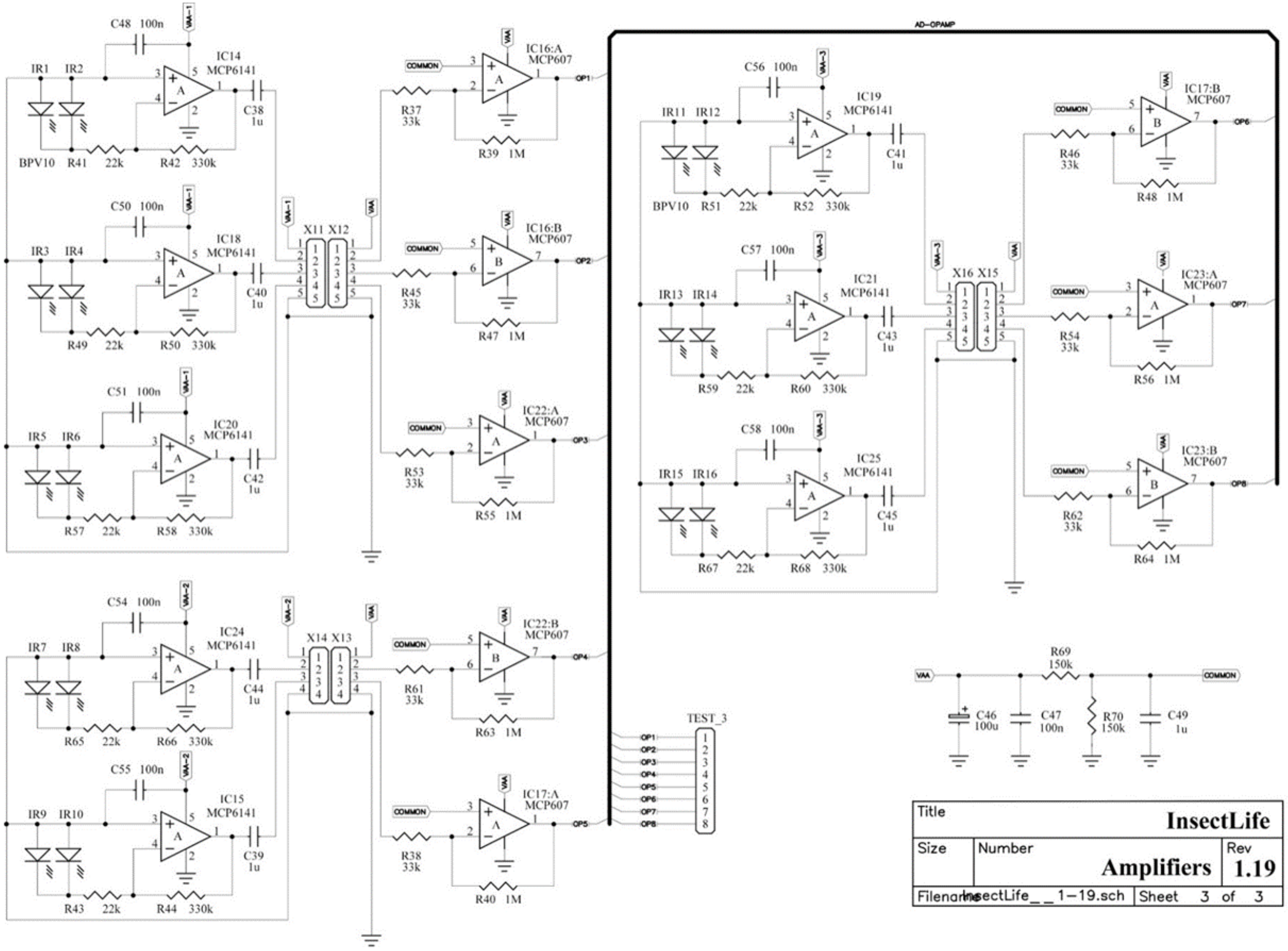

Figure 2. Photodiode receivers

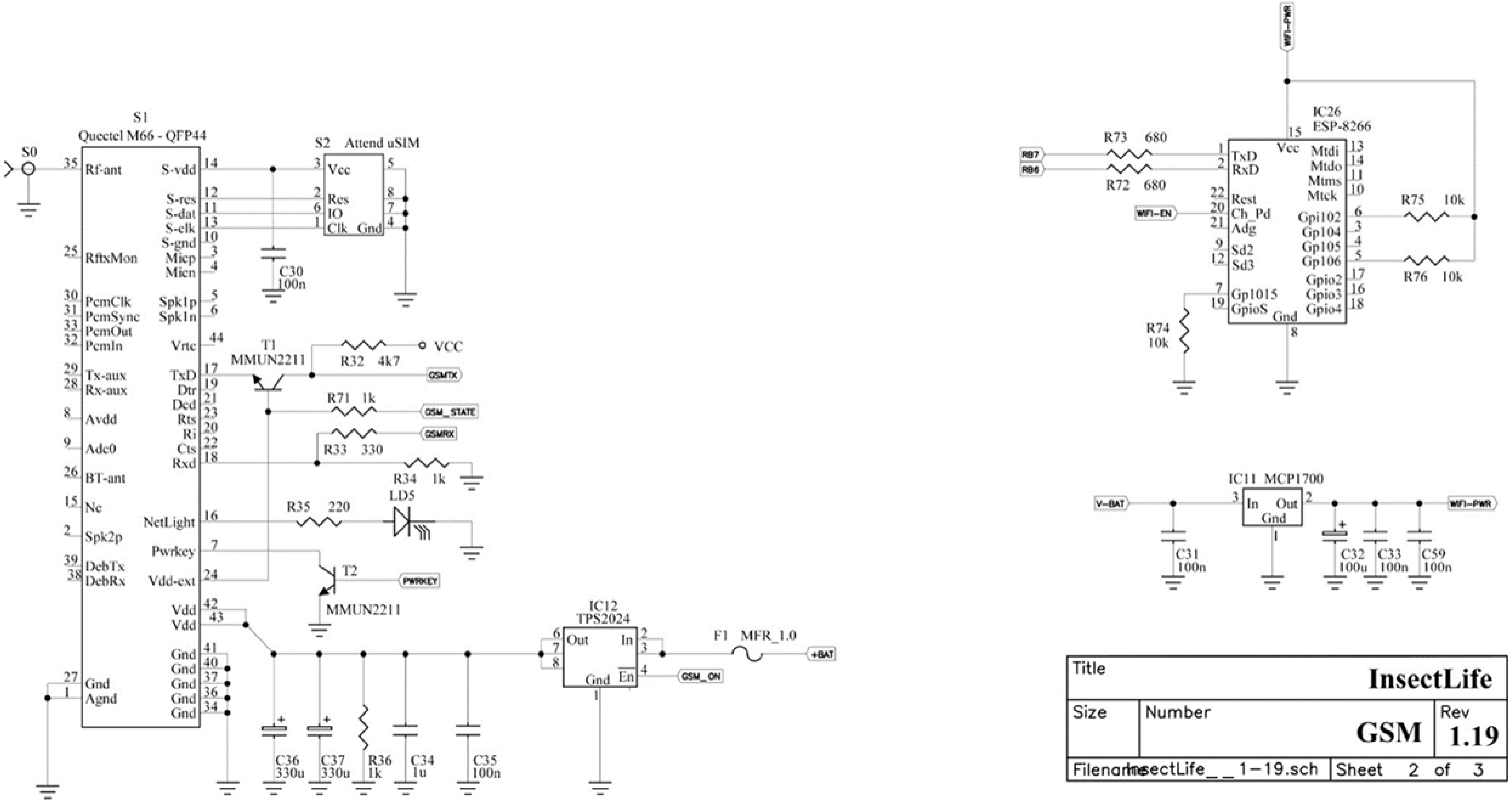

Figure 3. GSM communication unit

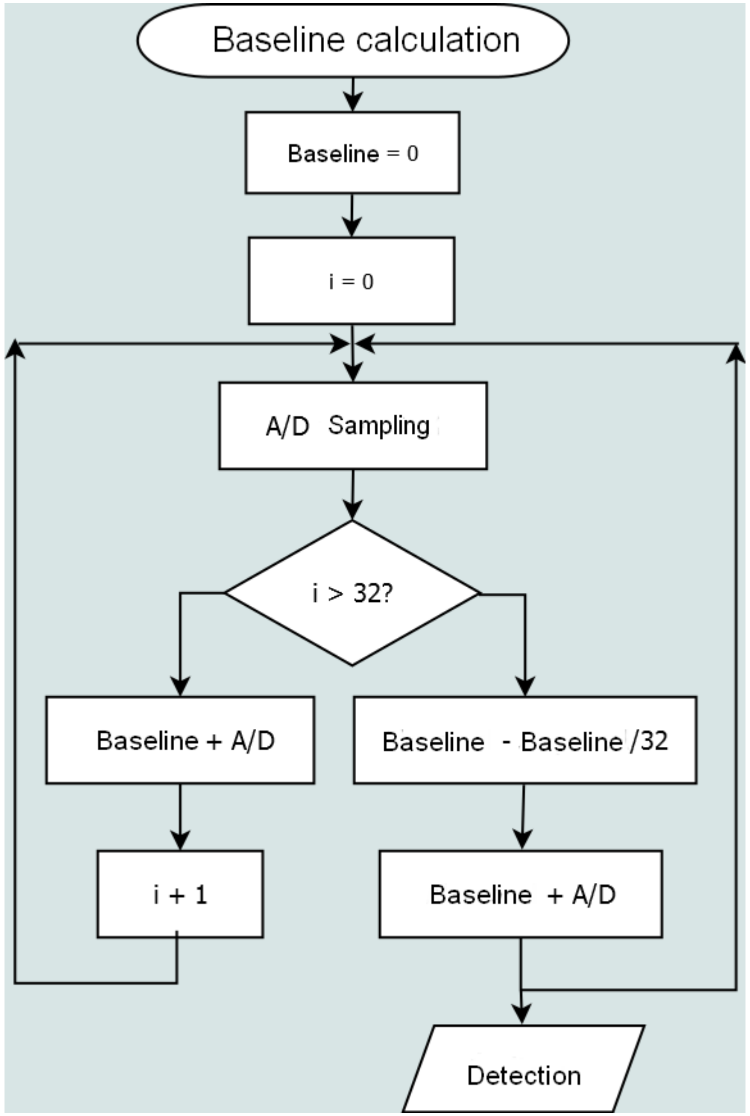

Figure 4. Flow chart of the baseline calculation and detection start

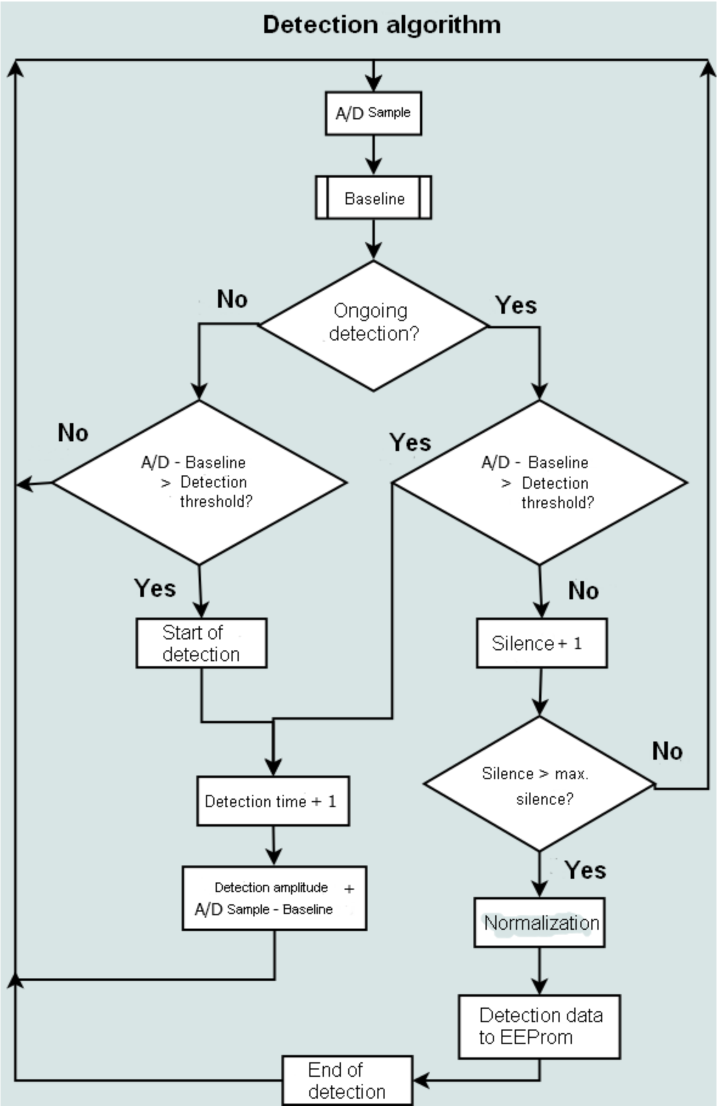

Figure 5. Flow chart of the detection algorithm
